# The Applicability of the Distribution Coefficient, *K*_D_, Based on Non-Aggregated Particulate Samples from Lakes with Low Suspended Solids Concentrations

**DOI:** 10.1371/journal.pone.0133069

**Published:** 2015-07-22

**Authors:** Aine Marie Gormley-Gallagher, Richard William Douglas, Brian Rippey

**Affiliations:** 1 School of Environmental Sciences, University of Ulster, Coleraine, N. Ireland, BT52 1SA, United Kingdom; 2 School of Applied Sciences, Cranfield University, Bedfordshire, MK43 0AL, United Kingdom; Institute of Tibetan Plateau Research, CHINA

## Abstract

Separate phases of metal partitioning behaviour in freshwater lakes that receive varying degrees of atmospheric contamination and have low concentrations of suspended solids were investigated to determine the applicability of the distribution coefficient, *K*_D_. Concentrations of Pb, Ni, Co, Cu, Cd, Cr, Hg and Mn were determined using a combination of filtration methods, bulk sample collection and digestion and Inductively Coupled Plasma-Mass Spectrometry (ICP-MS). Phytoplankton biomass, suspended solids concentrations and the organic content of the sediment were also analysed. By distinguishing between the phytoplankton and (inorganic) lake sediment, transient variations in *K*_D_ were observed. Suspended solids concentrations over the 6-month sampling campaign showed no correlation with the *K*_D_ (*n* = 15 for each metal, *p* > 0.05) for Mn (*r* ^2^ = 0.0063), Cu (*r* ^2^ = 0.0002, Cr (*r* ^2^ = 0.021), Ni (*r* ^2^ = 0.0023), Cd (*r* ^2^ = 0.00001), Co (*r* ^2^ = 0.096), Hg (*r* ^2^ = 0.116) or Pb (*r* ^2^ = 0.164). The results implied that colloidal matter had less opportunity to increase the dissolved (filter passing) fraction, which inhibited the spurious lowering of *K*_D_. The findings conform to the increasingly documented theory that the use of *K*_D_ in modelling may mask true information on metal partitioning behaviour. The root mean square error of prediction between the directly measured total metal concentrations and those modelled based on the separate phase fractions were ± 3.40, 0.06, 0.02, 0.03, 0.44, 484.31, 80.97 and 0.1 *μ*g/L for Pb, Cd, Mn, Cu, Hg, Ni, Cr and Co respectively. The magnitude of error suggests that the separate phase models for Mn and Cu can be used in distribution or partitioning models for these metals in lake water.

## Introduction

Colloidal matter in lakes is composed of clays, labile organic matter, hydrous metal oxides, and phytoplankton artefacts and the particles typically range in size from 0.1 *μ*m to 1.0 *μ*m in diameter [1]. Its size overlaps with fundamental biogeochemical phases of metal cycling in lakes; the dissolved and particulate phases. The dissolved metal fraction is generally defined as the metals passing through a 0.45 *μ*m filter [2, 3] and the particulate fraction is an aggregate of two or more properties, predominately the phytoplankton and lake sediment, which frequently range in diameter size from 0 *μ*m to 0.1 mm [4]. The influence of colloids on metal partitioning behaviour in lakes is therefore difficult to operationally define, which is of particular significance when calculating the distribution (or partition) coefficient, *K*_D_.

The distribution coefficient is the most common and simplest method of estimating the extent of contaminant retardation from particles to water [5]. It is calculated as the ratio between metals in the particulate and dissolved phases [6, 7] and is used widely in prioritizing site remediation and waste management decisions [8]. However, consensus is building that the use of *K*_D_ as a descriptor for metal partitioning between solids and water is probably unsuitable [9].

There are limited studies on *K*_D_ in natural aquatic environments [10], and those available often record great variations in *K*_D_ as a result of biogeochemical factors and limitations of measurement [11, 9, 12, 7]. There are two related problems with *K*_D_. Firstly, to collect a concentrated sample of particles in the water column, sediment traps are frequently used [13], which not only exposes the sample to potential metal contamination for longer, but also produces time-averaged values that are input to the *K*_D_ formula with other parameters not reflective of the same timeframe. The concentrations of metals in the top 5 cm of sediment from the lakebed are often used to overcome this [14]. However, as mentioned, the particulate phase typically represents aggregated phytoplankton and lake sediment, both of which can have contrasting metal affinities that are not static in time or space. This means that, by not distinguishing between the particulate fractions, *K*_D_ may have little power in estimating partitioning behaviour because there may be dissimilar mechanisms controlling the distribution between the phases.

Secondly, the presence of colloids in water with a high suspended solids (SS) mass can disguise the true distribution of metals, because as SS increases, so too does the concentration of colloidal matter, which typically increases dissolved (filtrate) metal concentrations [4]. Therefore, an increase in colloids with SS may appear to dilute the particulate phase, lowering the *K*_D_. This typically results in an inverse relationship between *K*_D_ and particle concentration, i.e. the particle concentration effect (PCE [15]). In recognition of such problems, Benoit et al. [16] suggests it would be valuable if more research was carried out on the behaviour of *K*_D_ in freshwater systems that have a markedly low SS (≈ 0.1 mg/L) where the influence of colloids is minimised. Yet the PCE has been observed under conditions whereby the concentrations of colloidal matter are eliminated [17].

What remains unknown is whether the predicted inverse relationship between *K*_D_ and particle concentration is still evident when the phytoplankton and lake sediment are measured individually (i.e. non-aggregated particulate samples) and then incorporated into the *K*_D_ formula. This should be investigated in freshwater systems with a low SS so to discount the influence of colloids. To address this, we set out to: (1) investigate lake systems with a considerably low SS mass; (2) calculate separately the phytoplankton and lake sediment fractions of the particulate phase in order to yield metal *K*_D_s that reflect transient conditions; and (3) examine the relationship of these *K*_D_ with SS.

## Methods

### Site descriptions

**Fig 1:**
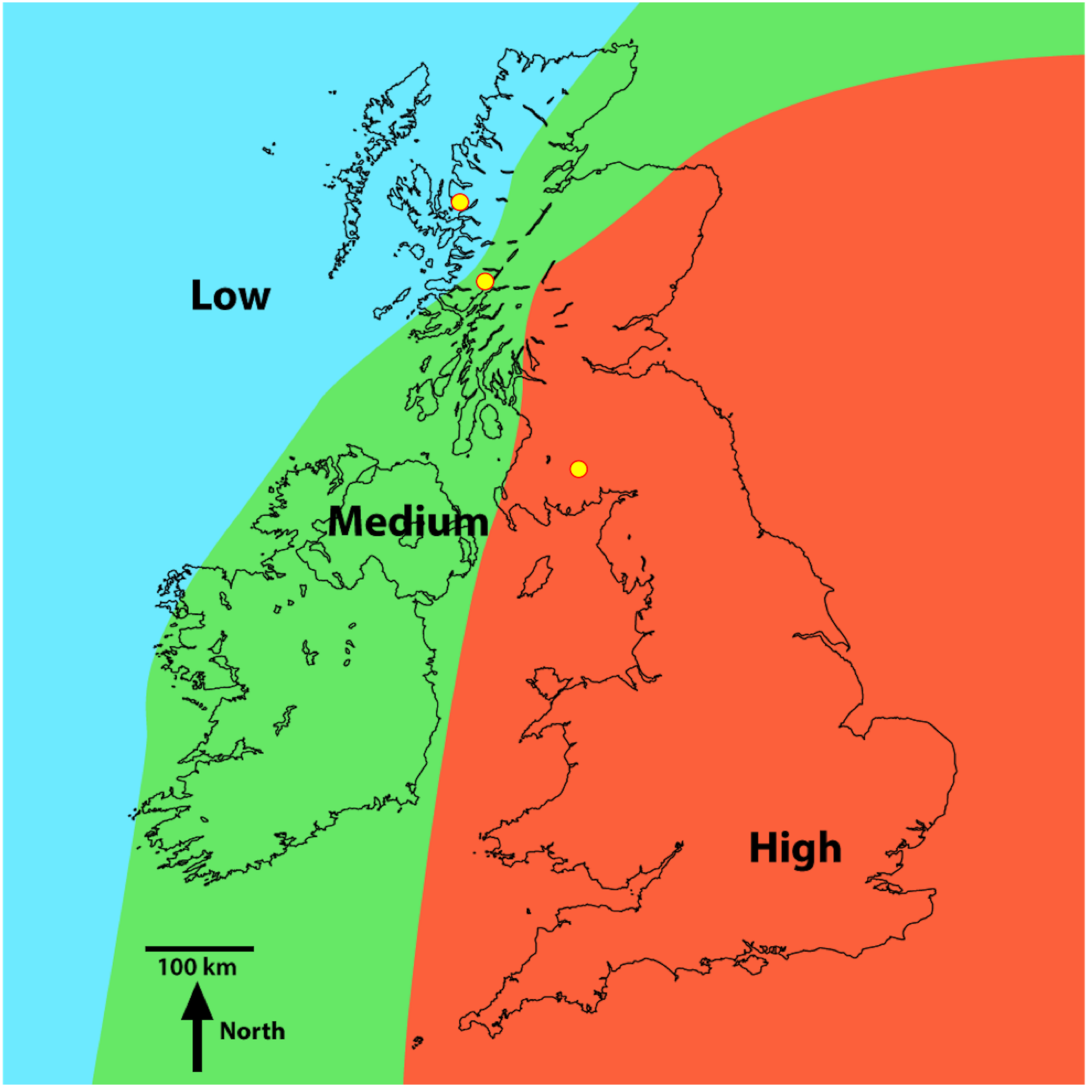
Three regions in the UK and Ireland of high, medium and low Pb contamination of lake sediment due to atmospheric deposition (from Rippey and Douglas, 2004). The study defined the regions by determining the metal burden from the background Pb concentrations (in lake sediment cores at depths dated pre-1900) and the Pb flux to the sediment at each core slice post-1900. The yellow circles in the low, medium and high regions of Pb contamination depicts the locations of Loch Coire nan Arr, Loch Doilet and Loch Urr, respectively.

Investigations were undertaken in three lakes that have been shown to receive varying degrees of metal contamination in the UK [18]. That is, one lake was selected in each of the regions that receive high, medium and low metal contamination from the atmosphere (Fig 1). Due to the need for appropriate lacustrine data on the relationship between metals in the phytoplankton and the dissolved phase [19, 20, 21, 22], it was considered important to obtain such data from a range of metal contaminated regions in order to address any variations. Also, the three lakes receive metal contamination solely from atmospheric deposition—and thus metal contamination from runoff or direct discharges would not influence our results [23, 18]. Additionally, the size and bathymetry of the lakes meant that regular sediment resuspension events (and by association high SS loads) would be unlikely to influence our investigation outcome [24, 25, 26].

In the following detailed site descriptions, lake surface area, perimeter, altitude, grid reference, catchment area and distance from the sea and to the nearest village were calculated and/or obtained using the OS Landranger Memory-Map V5 edition (2006) for northern and southern Scotland (Licence number PU 100034184). The maximum lake depths were based on collected field data, while catchment geology, vegetation and soil type were derived from Patrick et al. [27].

Loch Coire nan Arr has a surface area of 13.21 ha, a maximum lake depth of 11 m and a catchment area of 8.45 km^2^ (Table 1). It is the most northerly of the three sites and lies in the region of low metal contamination from the atmosphere [18]. The catchment is dominated by steep corrie cliffs, and the lake itself fills a large deep sandstone corrie that was carved by deglaciation at the end of the Pleistocene. Loch Coire nan Arr is one of the six UK sites represented in the UNECE International Co-operative programme on Assessment and Monitoring of Acidification of Rivers and Lakes [28]. Permission for sampling the site was obtained from The Applecross Trust, a conservation charity responsible for the management of the lake (contact: admin@applecross.org.uk).

**Table 1:** Summary of the site characteristics of Loch Coire nan Arr in northwestern Scotland, Loch Doilet in western Scotland and Loch Urr in southern Scotland.

	Loch Coire nan Arr	Loch Doilet	Loch Urr
**Grid Reference**	NG 808422	NM807677	NX759864
**Surface area**	13.21 ha	51.55 ha	47.0 ha
**Perimeter**	1.86 km	5.49 km	4.2 km
**Maximum lake depth**	11 m	16 m	13.2 m
**Lake volume**	5.6 x 10^5^ m^3^	4.1 x 10^6^ m^3^	2.35 x 10^6^ m^3^
**Distance upstream from sea**	2.03 km	6.2 km	22.7 km
**Aerial distance from nearest village**	8.91 km (Lochcarron)	8.84 km (Strontian)	6.6 km (Monaive)
**Elevation/altitude**	125 m	8 m	193 m
**Catchment area**	8.45 km^2^	33.51 km^2^	7.73 km^2^
**Catchment geology**	Torridonian Sandstone	Schists and gneiss	Granite / gneiss
**Catchment vegetation**	Confiers < 1%	Conifers—50%, moorland—50%	Moorland—100%
**Catchment soils**	Peat	Peats	Podsol, peaty gley blanket peat

Loch Doilet has a surface are of 51.55 ha, a maximum lake depth of 16 m and a catchment area of 33.51 km^2^ (Table 1). The lake, lying northwest of the Ben Nevis Mountain range, is the largest of the three lakes and has received moderate metal contamination from the atmosphere [18]. The catchment rises from the lake to a peak of approximately 720 m. The dominant soil types are peats, which are eroded on the uppermost reaches of the catchment [27]. Permission for sampling the site was obtained from the Forestry Commission Scotland, a UK non-ministerial government department responsible for the management of the lake (contact: lochaber@forestry.gsi.gov.uk).

Loch Urr has a surface area of 47 ha with a maximum lake depth of 13 m (Table 1). It lies in the Dumfries and Galloway region of south-west Scotland, an area that has received high metal contamination from the atmosphere [18]. The lake drains the smallest of the three catchments with an area of 7.73 km^2^. The underlying geology is mainly composed of granite / gneiss and the land-use is confined to low-intensity sheep grazing [27]. Permission for sampling the site was obtained from the Urr District Salmon Fisheries Board, a board of the Galloway Fisheries Trust charity set up to protect the lake and its catchment (contact: mail@gallowayfisheriestrust.org).

### Sampling

**Fig 2:**
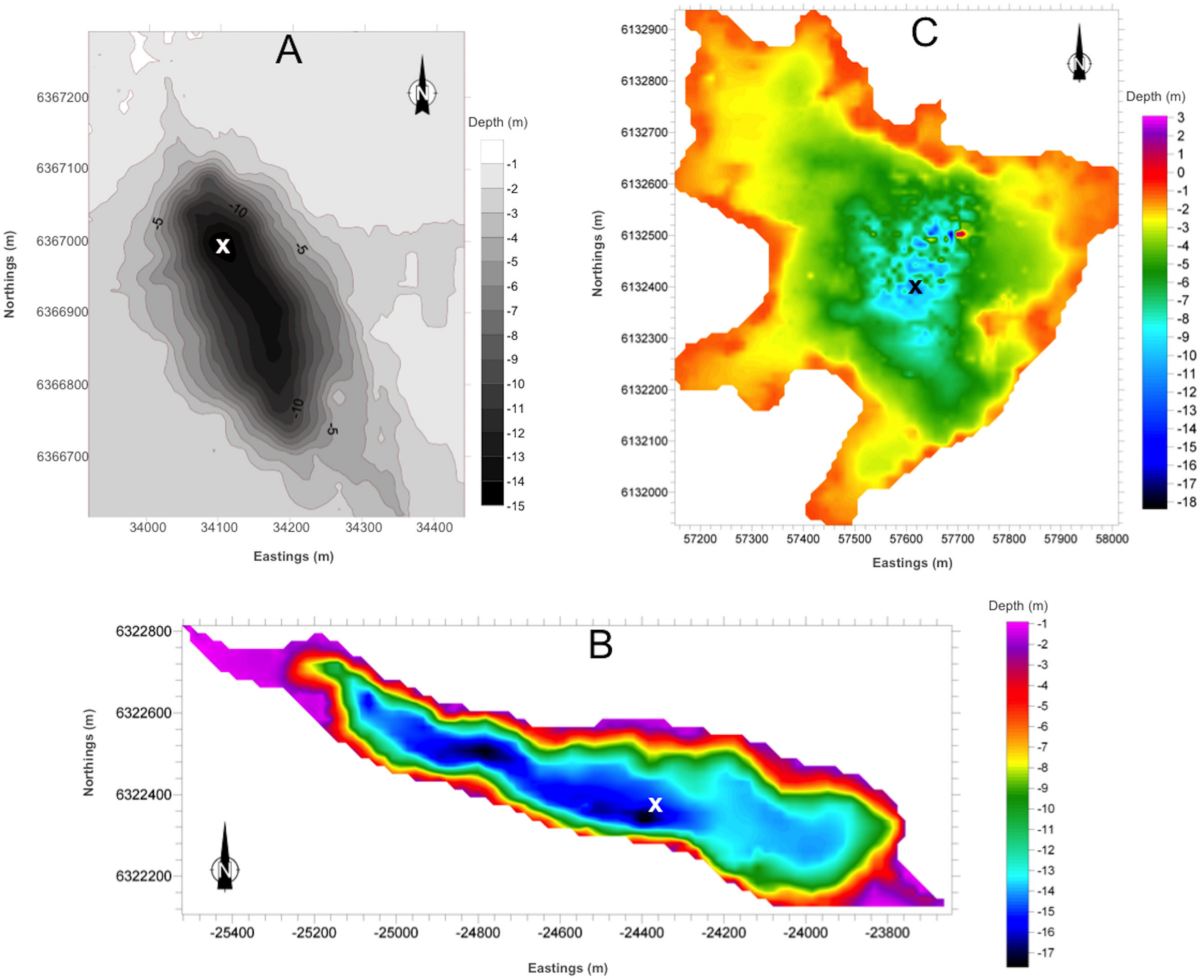
Hydrographic Charts of (A) Loch Coire nan Arr, (B) Loch Doilet and (C) Loch Urr. The hydrographic survey was completed with a MIDAS Surveyor GPS Echosounder supplied with a 210kHz transducer (Valeport, Devon, UK) at the beginning of this study. Approximate depth is shown in metres and a change in depth is indicated by the grey/colour scale. The X denotes where the sampling took place.

Sampling campaigns were conducted on five occasions: March, May, June, July and September 2007 at each of the three lakes. The deepest parts of the lakes were located for sampling using a LPS-1 Digital Handheld Depth Sounder (Vexilar USA) and hydrographic charts from a survey completed with a MIDAS Surveyor GPS Echosounder (Fig 2). Depth profiles of conductivity, temperature and dissolved oxygen were recorded at each sampling station with a YSI 556 Multi Probe System. Lake water was sampled from 6 m below the lake surface using a Perspex Ruttner sampler [29]. The reason for sampling at 6 m was to ensure sampling was taken from a consistent area where surface and bottom waters are completely mixed. Calculating the specific surface mixed layers was outside the scope of this study (see [30]). However, it has been previously shown that the free water surface over which wind acts upon in medium-sized lakes, such as those investigated in this study (according to, for example, Leppäranta’s [31] classification), is 6 m [32, 25, 33, 34].

Using the Perspex Ruttner sampler, on each sampling campaign at the three lakes, three 1 L water samples were collected for SS analysis and an additional twelve 50 ml water samples were extracted for metal, phytoplankton and chlorophyll *a* analysis. Below details the methods used to process the samples and determine metal content in the phases investigated. All equipment coming into contact with samples for metal analysis were prepared to reduce metal contamination and prevent adsorption losses [35]. For all phases, the XSeries^I^ ICP-MS (ThermoFisher Scientific) was used for the analysis of Pb, Cu, Co, Hg, Co and Mn in the samples. The precision of the laboratory analysis was assessed from triplicate analysis of reference material and field samples and accuracy by using blanks [36]. Additional methods for assessing error in calculations are detailed below.

### Phytoplankton analysis

At each lake, three 50 ml water samples were stored in vials pre-prepared with glutaraldehyde (50%, Electron Microscopy grade, EMS, U.S.A.) to produce a final concentration of 2% (v/v). A Nikon-5400 inverted light microscope at 40x magnification was used to identify, count and measure the phytoplankton, following the procedures described by Olrik et al. [37]. Cell counts were converted to counts per volume of lake water under the assumption that 1 mm^3^/L^-1^ was equivalent to 1 mg/L^-1^ wet weight [38]. Cell volumes and surface areas were calculated using the geometric equations of Hillebrand et al. [39]. The cell surface area and volume calculations were collated with cell counts per volume of lake water to equate the surface area and biomass per volume of lake water.

Net-haul phytoplankton samples were collected and acidified on-site using the approach set out by Ho et al. [40]. The samples were digested by treatment with hydrofluoric, nitric and perchloric acid (following Bock [41]). An empty beaker (a reagent blank) and two samples of certified reference material (Chinese stream sediment, GBW 07301, LGC Promochem, UK) were included with every batch. Metal concentrations in the standing crop of phytoplankton (M_*Phy*_, mg/L) were calculated through (Eq 1):

$${\text{M}}_{Phy}=Phy_{\text{Bulk}}\times \;\text{Biomass}$$ (1)

where *Phy*_Bulk_ is the concentration of metals per unit mass of phytoplankton (mg/kg), and Biomass is the phytoplankton biomass in the water column at the time of sampling (kg/L).

In addition, the concentration of the pigment chlorophyll *a* was determined in the water samples because it is an indicator of phytoplankton biomass [42]. The pigment was extracted from the filtered samples into 90% V/V methanol, and the detection was performed with a Shimadzu UV-Mini 1240 spectrophotometer set at an emission wavelength of 665 nm.

### Lake sediment analysis

A piston corer was used to extrude the upper 5-cm long of lakebed sediment [14]. The material was processed and analysed using the approach outlined for the phytoplankton material. The percentage weight loss on ignition (LOI) was determined on the sediment core sample following the methods set out by Heiri et al. [43].

For all sampling occasions at each lake, the 3 x 1 L water samples collected were used to gravimetrically determine SS by filtering through aliquots of known volume (1 L at a time) of lake water through oven-dried pre-weighted GF-C filter paper [44]. The concentration of metals in the SS (M_*S*ed_, mg/L) was determined through:

$${\text{M}}_{Sed}=\left[SS-\text{Biomass}]\;\times \;[Sed_{\text{Bulk}}-\left(\text{LOI}\;\times \;Sed_{\text{Bulk}}\right)\right]$$ (2)

where *SS* is the suspended solids concentration (kg/L), Biomass is as Eq 1 (kg/L), *Sed*_Bulk_ is the concentration of metals per unit mass of bulk sediment collected from the lakes (mg/kg), and LOI is the percentage weight lost on ignition of the sediment samples, which is proportional to the organic content of the samples (%).

### The dissolved phase

At each lake, water samples (plus blank samples of Millipore Milli-Q water) were acidified on-site in three 50 ml pre-prepared vials to 2% (w/v) HNO_3_^−^ (ARISTAR grade) following filtration through acid-washed 0.45 *μ*m filters. Metal concentrations were determined in the filtrate to represent the dissolved constituents [3].

### The partition coefficient

The partition coefficient *K*_D_ (l kg^-1^ dw^-1^) was calculated through Eq 3 [45].

$$K_{\text{D}}=\frac{C_{Part}÷SS}{C_{Diss}}$$ (3)

where *C*_*Part*_ is the particulate metal concentration (i.e. M_*Phy*_ + M_*Sed*_) in kg L^-1^, *C*_*Diss*_ is the dissolved (filter passing) concentration (kg L^-1^) and *SS* is the suspended solids concentration in mass dry weight (dw) per volume (kg dw L^-1^).

### Accuracy of using the separate phase fractions

The accuracy of using the separate phase fractions to model the particulate and dissolved metal concentrations was assessed by firstly measuring the metal content in (non-filtered) lake water samples. At each lake, three 50 ml water samples (plus blank samples of Millipore Milli-Q water) were acidified on-site to 2% (w/v) HNO_3_^−^ (ARISTAR grade) for the direct determination of total metal concentrations from spot samples.

Next, the modelled total metal concentrations were determined—representing lake sediment, phytoplankton and dissolved metal concentrations at a steady-state (Eq 4, mg/L).

$$C={\text{M}}_{Phy}+{\text{M}}_{Sed}+C_{Diss}$$ (4)

The errors between the modelled steady-state concentrations (Eq 4) and the measured concentrations were approximated by calculating the root mean square error of modelled concentrations with the difference in the measured and modelled concentrations, $[\surd \sum [{\left(modelled-measured\right)}^{2}]÷n]$. This method is used to assess the accuracy of the modelled values [46], although a separate data set would be necessary for an independent test of the model [47, 48].

Additionally, as a number of factors are included in the calculations of the total metal concentrations, the error in each of those factors were propagated through to the overall error in the modelled values. This was completed by calculation of the maximum (*C*_*Max*_) and minimum (*C*_*Max*_) total metal concentrations (Eq 5).

$$C_{Max}=\left[\left(SS_{Max}-{\text{Biomass}}_{Max}\right)\;\times \;\left(Sed_{\text{Bulk}}-\left(\text{LOI}\;\times \;Sed_{\text{Bulk}}\right)\right)\right]+{\text{M}}_{PhyMax}+C_{DissMax}$$ (5)

where *SS*_*Max*_ is the maximum suspended solids concentration (kg/L), Biomass_*Max*_ is the maximum biomass of phytoplankton in the water column at the time of sampling (kg/L), *Sed*_Bulk_ is as Eq 2, M_*PhyMax*_ is the maximum concentration of metals in the standing crop of phytoplankton in the water column (mg/L) and *C*_*Diss*Max_ is the maximum concentration of metals in the filtered water fraction (mg/L). Likewise, the minimum total metal concentrations were calculated according to the following Eq 6.

$$C_{Min}=\left[\left(SS_{Min}-{\text{Biomass}}_{Min}\right)\;\times \;\left(Sed_{\text{Bulk}}-\left(\text{LOI}\;\times \;Sed_{\text{Bulk}}\right)\right)\right]+{\text{M}}_{PhyMin}+C_{DissMin}$$ (6)

## Results and Discussion

**Fig 3:**
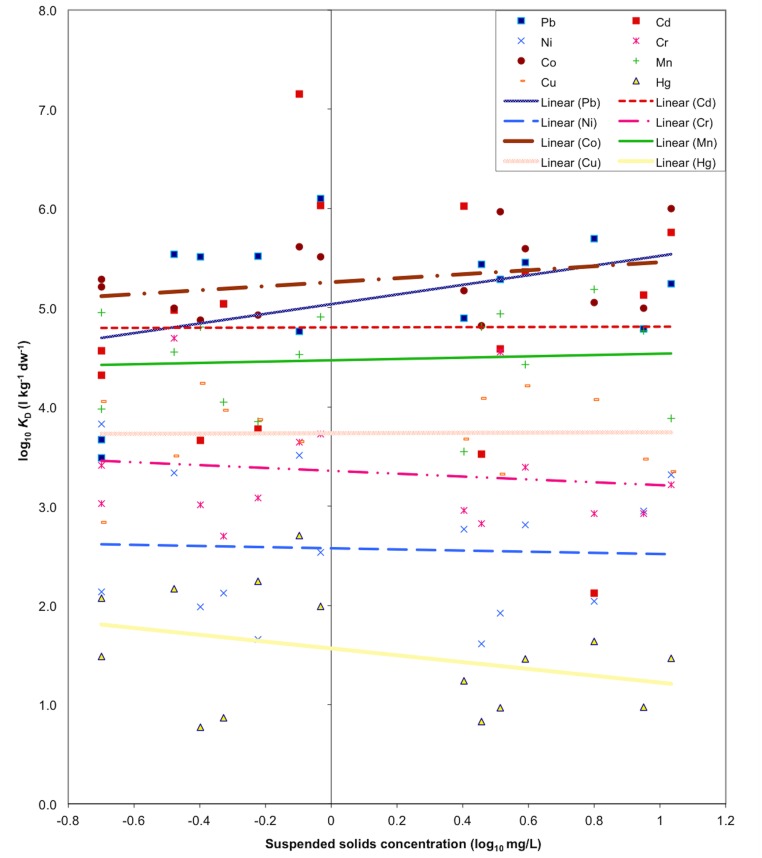
Correlation between the calculated *K*_D_ values for Pb, Ni, Co, Cu, Cd, Cr, Hg and Mn and the suspended solids concentration at the time of sampling (*n* = 15 for each metal, *p* > 0.05).

The suspended solids concentration at the time of sampling showed no correlation with the *K*_D_s (*n* = 15 for each metal) of Mn (*r* ^2^ = 0.0063), Cu (*r* ^2^ = 0.0002, Cr (*r* ^2^ = 0.021), Ni (*r* ^2^ = 0.0023), Cd (*r* ^2^ = 0.00001), Co (*r* ^2^ = 0.096), Hg (*r* ^2^ = 0.116) or Pb (*r* ^2^ = 0.164) (Fig 3). The significance of the regression analysis indicates that there was no relationship between the two variables (*p* > 0.05).

**Fig 4:**
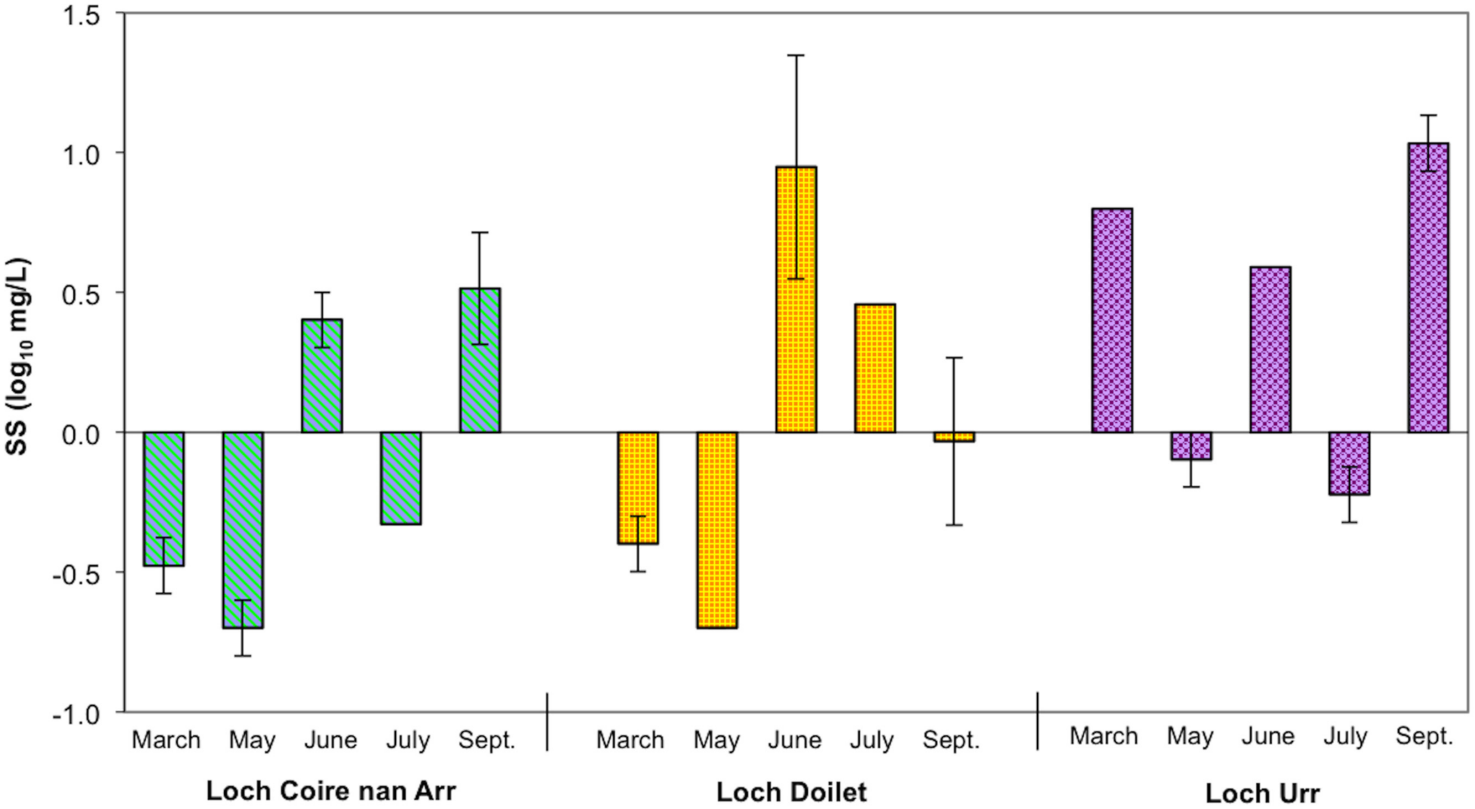
The SS concentration in Loch Coire nan Arr, Loch Doilet and Loch Urr during March, May, June, July and September 2007. Data are presented as the mean ± standard deviation (SD).

The results in Fig 3 conflict with reported patterns of *K*_D_ decrease with increasing SS due to the dilution of suspended matter and the resuspension of less contaminated sediment [6, 7, 5], i.e. the particle concentration effect (PCE [15]). As mentioned, the PCE can be attributed to colloidal matter in waters with a high SS range, which increases dissolved (filtrate) metal concentrations—lowering the *K*_D_ [49, 4]. In Benoit et al.’s study [16], they reported that as SS concentration declined, the rate of decrease in *K*_D_ with SS slowed down to the point that *K*_D_ became near constant when the SS concentration was low at around 1 mg/L. It is also notable that the SS range in this study, i.e. 0.2–10.8 mg/L (Fig 4), was an order of magnitude lower than that investigated by Benoit et al. (i.e. 1–100 mg/L).

These results can be understood further by examining the calculations behind the relationship of *K*_D_ and SS. The concentration of SS is inevitably included in the determination of particulate metals in the water column. The association between *K*_D_ and SS may then be viewed as a spurious correlation because, as Eq 7 demonstrates, if the logarithms are taken of both sides on Eq 3 and are divided by log(SS), we get [45]:

$$\frac{\text{log}\left(K_{\text{D}}\right)}{\text{log}\left(SS\right)}\;=\;\frac{\text{log}\left(C_{Part}\right)}{\text{log}\left(SS\right)}\;-\;\frac{\text{log}\left(C_{Diss}\right)}{\text{log}\left(SS\right)}\;-\;1$$ (7)

It can be ascertained from Eq 7 that the slope of the correlation between log(*K*_D_) and log(*SS*) is -1. Considering this spurious slope, *K*_D_ values would decline with the higher (supposedly) dissolved fraction. This inevitable correlation may be particularly noticeable in samples containing high concentrations of colloids because this can increase filtrate metal concentrations and thus increase the denominator in Eq 3. This is in agreement with Hart and Lowery [50] who warned that because a high colloidal fraction decreases the highest values of *K*_D_, particle concentration has more influence on *K*_D_. Lindstrom [51] also recommends that the use of *K*_D_ in modelling is discontinued because the ratio that includes SS concentrations is greatly affected by spurious correlations and that the particulate fraction (*C*_part_: total metal) be used as a simple alternative.

The potential influence of colloids on the behaviour of *K*_D_ is also manifested in the differences between the mean *K*_D_ of the metals (Fig 3). The mean *K*_D_ (l kg^-1^ dw^-1^) of the metals ranges in the order of (*n* = 15 for each metal) Cd (1.2 x 10^6^) > Pb (2.7 x 10^5^) > Co (1.9 x 10^5^) >Mn (3.1 x 10^4^) > Cu (3.2 x 10^3^) > Cr (2.2 x 10^3^) > Ni (1.2 x 10^3^) > Hg (82.0). In theory this suggests the high to low range of *K*_D_s corresponds to a decreasing reactivity with particulate matter [52]. However, as previously described, the *K*_D_ values may be lowered due to a colloidal matter increase. It may be more feasible that the variations across the mean *K*_D_s of the metals relate to the tendency of each metal to form organic complexes [15, 1]. For example, it has been observed that organic Co complexes in natural waters are weak and relatively less abundant than the organic complexes of Ni [53, 54]. Therefore, Co has a higher mean *K*_D_ than Ni, as observed here, because of the relatively low influence of colloidal Co interactions.

The results in Fig 4 also highlight reproducibility errors in the SS measurements. This could be because the low SS concentrations meant that some of the measurements are near/below the limit of detection of the published method, i.e. 0.5 mg/L [55, 56]. As SS concentrations are included in *K*_D_ calculations, this highlights an important related problem—that *K*_D_ is difficult to measure not only in higher SS lakes due to the enhanced problem of colloids as described, but also in low SS lakes due to the limitations of measurement using standard methods. Another interpretation relates to biogeochemical factors. On the occasions with high reproducibility error—firstly Loch Doilet in June, this particular sampling date was an extremely windy occasion and the lake showed its highest dissolved oxygen (12.0 mg/L) and conductivity (24.2 ppm) readings. This suggests the variation might be related to Hilton’s processes in a lake [24, 25, 26]. Regarding all three lakes in September, Loch Urr and Loch Doilet had its highest chlorophyll *a* (23 *μ*g/L and 2.7 *μ*g/L respectively), while Loch Coire nan Arr had its second highest chlorophyll *a* reading (3.3 *μ*g/L)–suggesting that productivity could influence the variation. This means that SS may be higher/lower at times because of other factors (apart from methods)–which inevitably affects the *K*_D_s.

It is also important to point out that if the SS data with poor reproducibility were omitted on this basis, there would be little or no effect on the findings depicted in Fig 3. That is, on excluding the three data with the highest SD (Loch Doilet in June, and Loch Coire nan Arr and Loch Urr in September), SS concentrations over the sampling campaign still show no correlation with the *K*_D_ (n = 12 for each metal in this case, *p* > 0.05) for Mn (*r* ^2^ = 0.022), Cu (*r* ^2^ = 0.198, Cr (*r* ^2^ = 0.078), Ni (*r* ^2^ = 0.074), Cd (*r* ^2^ = 0.026), Co (*r* ^2^ = 0.004), Hg (*r* ^2^ = 0.046) or Pb (*r* ^2^ = 0.299). Furthermore, when we performed this test also omitting September’s result for Loch Urr, there remains no correlation between the SS and *K*_D_ (n = 11 for each metal in this case, *p* > 0.05) for Mn (*r* ^2^ = 0.022), Cu (*r* ^2^ = 0.20, Cr (*r* ^2^ = 0.085), Ni (*r* ^2^ = 0.073), Cd (*r* ^2^ = 0.029), Co (*r* ^2^ = 0.005), Hg (*r* ^2^ = 0.049) or Pb (*r* ^2^ = 0.3).

**Fig 5:**
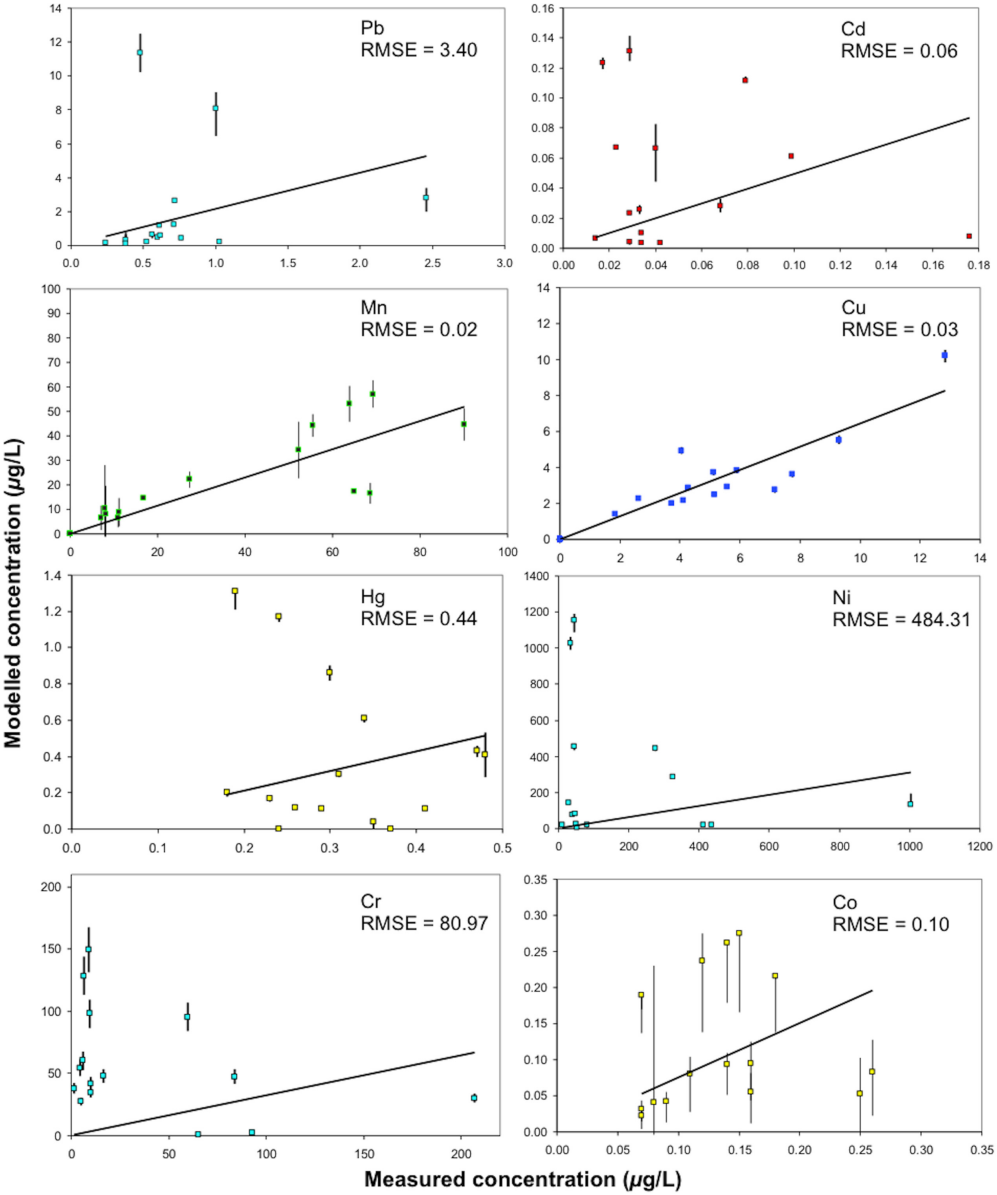
Modelled total Pb, Ni, Co, Cu, Cd, Cr, Hg and Mn concentrations based on the phytoplankton, lake sediment and dissolved phases (using Eq 3) versus the directly measured total metal concentrations. The 1 to 1 line, maximum and minimum modelled concentration due to propagation of the maximum and minimum concentrations in each phase, and the root mean square error of model prediction (RMSE, *μ*g L^-1^) are shown.

The totals (in *μ*g/L) of the separate phase fractions (i.e. those modelled based on Eq 4) are compared against the concentrations determined in the spot samples taken at the time of sampling in Fig 5. The model errors are ± 3.40, 0.06, 0.02, 0.03, 0.44, 484.31, 80.97 and 0.1 *μ*g/L for Pb, Cd, Mn, Cu, Hg, Ni, Cr and Co respectively. When the ratio of model error to the maximum concentration measured is used to determine the relative performance of the model [48], it performs from best to worst in the following order; Cu (≈ 0.0003), Mn (≈ 0.0002), Pb (≈ 0.299), Co (≈ 0.327), Hg (≈ 0.379), Ni (≈ 0.42), Cd (≈ 0.483) and Cr (≈ 0.542).

In theory, Fig 5 suggests that Cu and Mn behaved the most conservatively in the lakes. To suggest a metal behaves conservatively in the context of the model used here (Eq 4) means that changes in the distribution between the phases are accounted for in the mixed phase total [57, 58]. The apparent conservative behaviour of Mn and Cu has been exhibited elsewhere. For example, it has been noted that, Mn in its carbonate form MnCO_3_ (rhodochrosite) is not involved in oxidation-reduction reactions and can therefore behave more conservatively than, for example, Co [59, 60]. In the case of Cu, the high level of Cu complexation to ligands present in natural waters can enhance the resistance of Cu to changes in pH [61, 62].

There are, however, four main elements of uncertainty to this interpretation. Firstly, it is possible that the modelled (Eq 4) and directly measured total metal concentrations are similar because the filtered metal fraction used to obtain the modelled values was determined from the filtrate of the same lake water used to obtain the measured values. Secondly, the dissolved (filtrate) fraction is likely to represent a greater a degree of contamination than the other fractions because phytoplankton and lake sediment measurements were based on concentrated samples and then calculated down to the specific fraction they represent. Thirdly, it is important to note that the data is based on samples taken from the deepest area of the lakes—and these are assumed to represent the whole lake. This means that the degree of sediment focusing is not accounted for [24], that spot samples of lake water can represent the SS, phytoplankton and total metal content in the lakes, and that the top 5 cm of lakebed sediment represented (minus the organic content) the metal concentrations in the SS. Fourthly, due to the size range of colloidal matter potentially overlapping with the other phases in biogeochemical cycles [1, 4], it is operationally difficult to distinguish between the phases and, therefore, any result inevitably reflects some concentrations in two or more phases. Nevertheless, the correlation of the modelled and measured Mn and Cu values in Fig 5 cannot be ruled out as a reflection of conservative behaviour as the analysis was handled in an identical manner for the three lakes and thus useful comparisons can be made.

## Conclusion

The absent correlation between the *K*_D_ of each metal and SS does not support the PCE theory. Imperative to our interpretation was the considerably low concentration range of SS in the three lakes during this investigation. This meant colloidal matter had less opportunity to increase the dissolved (filter passing) fraction, which inhibited the spurious lowering of the *K*_D_s and subsequent PCE. These findings conform to the increasingly documented theory that the use of *K*_D_ in modelling may mask true information about metal partitioning behaviour.

Based on a simple mixing model used to estimate the particulate and dissolved Cu, Mn, Pb, Co, Hg, Ni, Cd and Cr concentrations, Cu and Mn appeared to behave the most conservatively in the lakes. This is in agreement with reports on the stability of Cu and Mn in the literature. Interpretation of these findings is limited by a potential degree of contamination in the filtrate samples compared to the measurements derived from bulk samples, inherent difficulties in operationally defining each phase and underlying assumptions that the samples taken represent the whole lake. The magnitude of the errors in relation to the ranges of concentration suggests that the separate phase models for Mn and Cu can be used in distribution or partitioning models for these metals in lake water.
